# Computational modeling investigation of pulsed high peak power microwaves and the potential for traumatic brain injury

**DOI:** 10.1126/sciadv.abd8405

**Published:** 2021-10-29

**Authors:** Amy M. Dagro, Justin W. Wilkerson, Thaddeus P. Thomas, Benjamin T. Kalinosky, Jason A. Payne

**Affiliations:** 1 U.S. Army Research Laboratory, Aberdeen Proving Ground, MD 21005, USA.; 2 J. Mike ‘66 Department of Mechanical Engineering, Texas A&M University, College Station, TX 77843, USA.; 3 General Dynamics Information Technology, JBSA Fort Sam Houston, San Antonio, TX 78234, USA.; 4 Air Force Research Laboratory, 711th Human Performance Wing, Airman Systems Directorate, Bioeffects Division, Radio Frequency Bioeffects Branch, JBSA Fort Sam Houston, San Antonio, TX 78234, USA.

## Abstract

When considering safety standards for human exposure to radiofrequency (RF) and microwave energy, the dominant concerns pertain to a thermal effect. However, in the case of high-power pulsed RF/microwave energy, a rapid thermal expansion can lead to stress waves within the body. In this study, a computational model is used to estimate the temperature profile in the human brain resulting from exposure to various RF/microwave incident field parameters. The temperatures are subsequently used to simulate the resulting mechanical response of the brain. Our simulations show that, for certain extremely high-power microwave exposures (permissible by current safety standards), very high stresses may occur within the brain that may have implications for neuropathological effects. Although the required power densities are orders of magnitude larger than most real-world exposure conditions, they can be achieved with devices meant to emit high-power electromagnetic pulses in military and research applications.

## Teaser

Simulations show that extremely high-powered microwave pulses can cause potentially injurious intracranial stresses.

## INTRODUCTION

The interactions between biological effects and electromagnetic (EM) fields (EMFs) are the subject of over a century of scientific research, motivated by innovations in biomedical applications and the development of safety standards. For EMFs in the frequency range of 0 Hz to 300 GHz, the most widely used guidelines for human exposure are designed to protect against adverse health effects associated with electrostimulation as well as local and whole-body heating (*1*). For frequencies in the radiofrequency (RF)/microwave range, it is well accepted that tissue heating is typically the main effect from interactions with EMFs (*2*–*4*).

The bulk of scientific literature uses continuous waves and moderate field strengths (typical of real-life scenarios), with less emphasis on pulsed fields of very high peak strength that may occur with ultrawideband pulse generators or EM pulse simulators (*4*). It is worth investigating whether extremely high peak power sources applied with a slow repetition frequency, or low duty cycle, can induce injurious effects without thermal buildup greater than a few degrees Celsius. Previous studies suggest that some nonthermal effects, such as electroporation, can only occur at extremely high thresholds. For example, membrane rupture from electroporation typically requires field strengths within tissues to exceed ~50 to 100 kV/m applied for pulse durations of >100 ns (*4*–*6*). Another example of a known nonthermal effect is the formation of thermoelastic stress waves that are attributed to small (but rapid) increases of temperature. The potential of these stress waves to initiate injury mechanisms may warrant further investigation. The thermoelastic stress wave mechanism is exemplified by the well-known microwave auditory effect (MAE).

The MAE, also referred to as “microwave hearing” or the “Frey effect” due to its discovery by Allan Frey in 1961 (*7*, *8*), was initially observed when subjects standing up to hundreds of feet away from a radar transponder could hear an audible tonal noise (e.g., chirping, buzzing, or clicking). The scientific underpinnings of the MAE were controversial for the first several years (*9*–*11*). After more than a decade of investigations, it became generally accepted that the perceived sound is due to the cochlea detecting stress waves that result from a rapid temperature rise in tissues within the head due to pulsed RF/microwave exposure (*11*, *12*). As the intracranial contents undergo a small and rapid thermal expansion from the absorbed EM energy, stress waves are generated and reflected within the head at a frequency that can be detected by the sensitive hair cells of the cochlea. The cochlea then relays the detected frequencies to the central auditory system, similar to the process involved in normal hearing. The difference is that “hearing” the MAE sound does not require air pressure waves to be transmitted from the ear canal to the inner ear.

Typically, relatively low-average powers and small temperature changes (10^−6^°C) are required to elicit the MAE (*12*). Although adverse health effects from the MAE have not been previously established, one study on rodents suggests that very high–peak power pulsed microwaves can result in cognitive deficits (*13*). Because these findings have not been replicated, it remains controversial whether high peak power microwaves can cause cognitive effects at exposure levels below established thermal thresholds. These previous findings, combined with advances of high-power microwave (HPM) technology in recent years, raises the question, can high peak power pulsed microwaves cause adverse health effects in addition to the MAE?

**Fig. 1.**
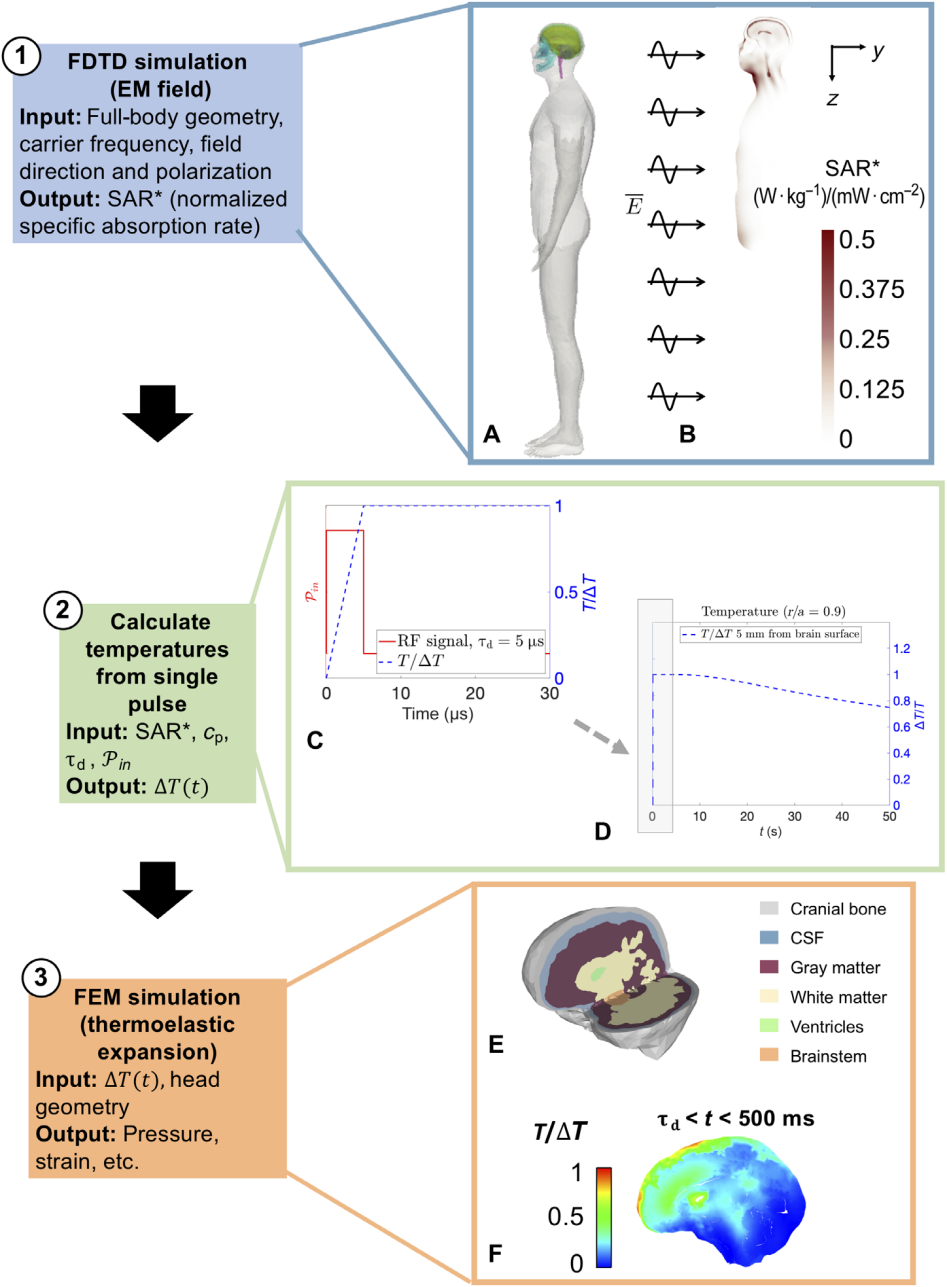
Flow chart of the computational approach. First, a simulation of full-body irradiation with microwaves (**A** and **B**) is used to find the normalized SAR (SAR*) within tissues. The SAR* values are used to compute temperature changes (**C**) and registered as initial conditions to a 3D FEM. At the end of the pulse duration (e.g., τ_d_ = 5 μ*s*), the temperatures are maintained constant in the FEM simulations because of the slow time scales of thermal conduction, as shown by the idealized approximation of cooling at the surface from a single pulse in (**D**). The FEM (**E**) is prescribed as temperature initial conditions for computation of the early time (<500 ms) mechanical response (**F**).

This study uses a two-simulation approach to investigate whether an HPM source could theoretically induce adverse mechanical responses within the brain. The first simulation uses the finite-difference time-domain (FDTD) technique to obtain the normalized specific absorption rate (SAR*) profile within a three-dimensional (3D) full-body human model resulting from EM plane waves of various RFs and incident angles (Fig. 1, A and B). The SAR values are then used to calculate changes in temperature throughout the head by assuming a linear increase in temperature during a single RF pulse. Next, these temperature changes are applied as initial conditions in a second simulation that uses a finite element model (FEM) (Fig. 1, C and D) to calculate the mechanical response of brain tissue in a realistic human head geometry at short time scales. Previous numerical studies have used a similar computational approach to study the MAE (*14*); however, these studies focused on the resulting acoustic emission frequencies and not the consideration of traumatic brain injury (TBI) due to the mechanical response following intense microwave exposures. Here, we compare our simulation results to previously established mechanics-based injury thresholds for strain and tensile pressure (i.e., cavitation). We examine the effects of pulse duration, incident power density, and EM field direction on the resulting tensile pressures in the simulation. The results of this study may guide empirical efforts investigating the biological effects of high peak power RF exposures, particularly for the types of exposure conditions where cognitive effects have been previously observed but never replicated.

## RESULTS

### Temperature distributions from RF exposure and frequencies of interest

The FDTD method was used to calculate the normalized SAR (denoted here as SAR*) within a simplified 3D human body model [see Materials and Methods and (*15*) for details], where SAR is a commonly used dosimetric quantity that is defined as the time derivative of incremental energy absorbed/dissipated by an incremental mass contained in a volume of a given density (*16*). Units for SAR are typically expressed in watts per kilogram. Details on the FDTD method are presented in Materials and Methods.

**Fig. 2.**
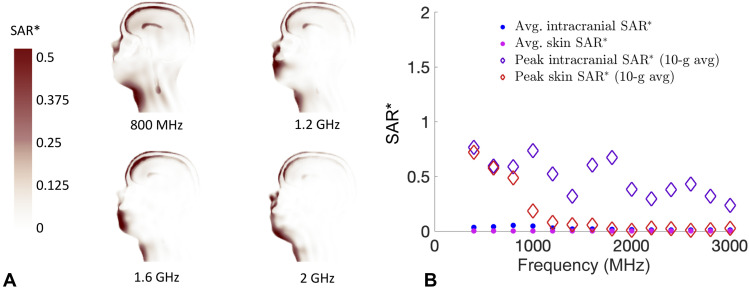
Calculation of SAR values from EM modeling. (**A**) Cross-sectional views of the SAR* values calculated from the FDTD simulations of 800, 1200, 1600, and 2000 MHz. (**B**) Comparison of average SAR* and peak 10-g averaged SAR* values in skin versus intracranial contents.

Simulated SAR* data at 800, 1200, 1600, and 2000 MHz are shown as cross-sectional images in Fig. 2A, where the values for SAR* are normalized by an incident power density with units of mW/cm^2^ (units of SAR* are (W · kg^–1^)/(mW · cm^−2^). For short time scales (<1 ms), we can ignore the effects of thermal diffusivity (*17*), and the initial rise in temperature is related to the SAR through the following relationship

$$\text{SAR}=\frac{c_{p}\Delta T}{\tau_{d}}=P_{IN}\times {\text{SAR}}^{*}$$ (1)

where *c*_p_ is the specific heat capacity of the tissue, ∆*T* is the temperature change, and τ_d_ denotes the pulse duration (*18*). Using the known values of specific heat capacity of various tissues, SAR* can be scaled by an applied incident power density $P_{IN}$ to calculate the change in tissue temperature.

Simulations are performed of an EM plane wave propagating toward the front of a 3D human body (electric field polarization parallel to the long axis of the body) at 14 different RFs between 400 and 3000 MHz. The incident EM wave is prescribed a vertical electric field polarization. For the frequency range in this study, it is observed that the EM energy is primarily deposited near the skin at frequencies of >2 GHz (Fig. 2A). Figure 2B shows a comparison of the mean SAR* and peak-localized SAR* (10-g averaged) values in the intracranial contents [white matter, gray matter, and cerebrospinal fluid (CSF)] versus the skin (from the head). The peak-localized SAR* values are calculated as the maximum 10-g averaged SAR*, in accordance with the Institute of Electrical and Electronics Engineers (IEEE) C95.1 RF exposure guidelines. With the exception of low intracranial absorption at 1400 MHz, the highest ratio of peak average intracranial SAR* to peak average skin SAR* occurs between 1 to 1.8 GHz. In an effort to maximize the effects of thermoelastic expansion, it is important to consider both the maximum temperature and the volumetric amount of brain tissue that is elevated. As an illustrative example in this work, we will present simulation results for a 1-GHz exposure, but note that similar temperature distributions can be achieved with most frequencies between 0.8 and 2 GHz.

### Simulated pressure fields induced by a single intense microwave pulse at 1 GHz

**Fig. 3.**
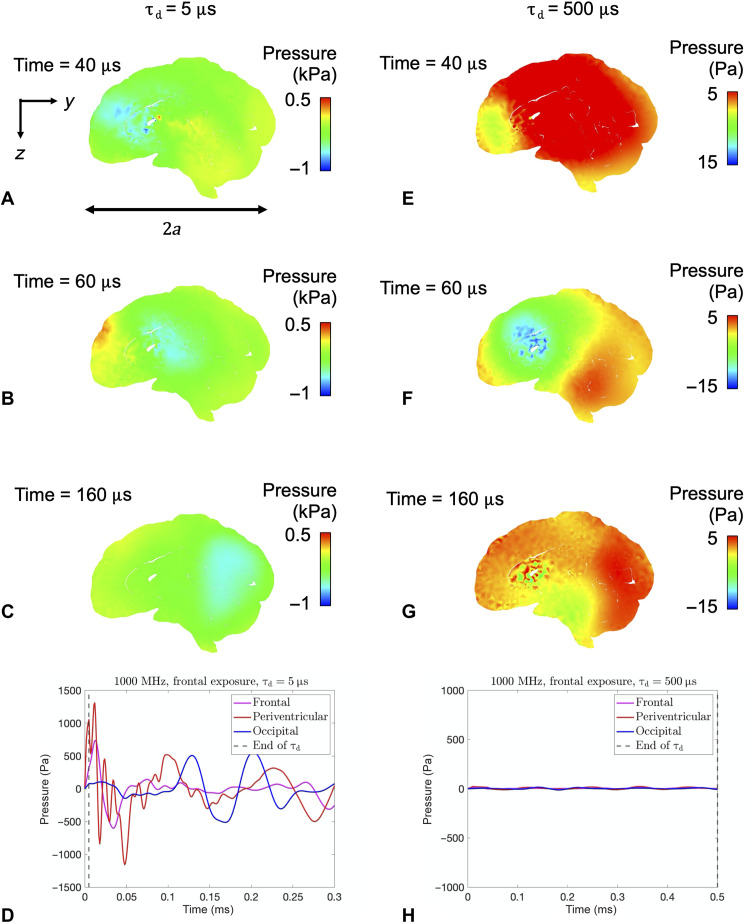
Comparison of pressure histories for different pulse durations. Pressure histories for τ_d_ = 5 μs (**A** to **D**) and τ_d_ = 500 μs (**E** to **H**) for an applied frequency of 1 GHz (power densities of 1 × 10^6^ mW/cm^2^ and 1 × 10^4^ mW/cm^2^ corresponding to 0.001°C peak localized intracranial temperature for τ_d_ = 5 μs and τ_d_ = 500 μs, respectively). Panels (D) and (H) show the pressure histories for three locations over time (single points located at the frontal lobe, periventricular region, and the occipital lobe).

The pressure fields induced in the brain tissue following a pulse of microwave energy are known to depend on both the power density and duration of the applied pulse. As described in more detail in Materials and Methods, we consider power densities on the order of 1 × 10^7^ W/m^2^ (1 kW/cm^2^) to be an example of a possible exposure condition for pulses in the microsecond regime. Because it is easier (with respect to required power density) to achieve higher intracranial temperatures with longer pulse durations, the effect of increasing the pulse duration on the mechanical response is examined here. To understand the effects of pulse duration on the stresses generated during rapid thermoelastic expansion, we can define an intrinsic time scale of a spherical geometry, τ_c_, where τ_c_ = *a*/*c*_B_ for radius *a* and elastic bulk wave speed *c*_B_. Our previous work shows that, for a spherical geometry, the ratio of pulse duration to this intrinsic time scale, τ_d_/τ_c_, determines the extent of “stress focusing” of the thermally induced stress waves. The material closer to the brain center is inertially confined, which leads to constrained thermal expansion and induces thermoelastic stresses. Because of the spherical-like geometry of the head, stress waves converge near the deep regions of the brain to create high tensile stresses (stress focusing). Approximating the head as a spherical geometry and setting 2*a* = 0.17 m (the anterior-to-posterior length of the head in the model) with *c*_B_ = 1450 m/s [roughly the bulk wave speed of brain tissue (*19*)], the characteristic time scale is approximately τ_c_ = 59 μs. Informed by these results from a spherical geometry, here, we conducted simulations in the realistic head models for two different pulse durations: τ_d_ = 5 μs ≪ τ_c_ and τ_d_ = 500 μs ≫ τ_c_. A comparison of the pressure histories in the human head for τ_d_ = 5 μs and τ_d_ = 500 μs is shown in Fig. 3 (A to D) and Fig. 3 (E to H), respectively.

For the case of τ_d_ = 5 μs, stress waves interact and create large tensile stresses in the frontal, periventricular, and occipital regions of the brain at 40, 60, and 160 μs, respectively (Fig. 3, A to C). Because of the asymmetric thermal load on the brain geometry, the focused tensile region moves from the front of the brain to the back of the brain in 2τ_c_. A pressure history plot for these three regions is shown in Fig. 3D.

For cases where τ_d_ >> τ_c_, it is expected that the magnitude of stress focusing will be smaller as shown in Fig. 3 (E to H). In the case of τ_d_ = 500 μs, most brain tissue will not exceed the cavitation threshold, even for these extreme energy densities. Because the loading time is much longer than the time it takes for the wave to traverse from the front of the head to the back of the head (∼160 μs), the stresses have undergone reflections by the end of the pulse duration, and there is enough time for the stresses to equilibrate.

Under equilibrium loading, thermal stresses are generated if and only if there is either (i) confinement of the body or (ii) an incompatible thermal strain field, i.e., **∇ × ∇ × α**∆T ≠ **0**, with **α** as the linear coefficient of thermal expansion, see Ravaji *et al.* (*20*). Because the CSF applies little to no confinement to brain deformation, here, equilibrium thermal stresses are generated only from thermal strain incompatibilities, which are present because of the nonlinear spatial variation of the temperature field as shown in Fig. 1F. Under equilibrium conditions, a nonzero stress field is required to enforce total strain compatibility, i.e., **∇ × ∇ × ε** = **0,** where **ε = C**^**−1**^**σ + α**∆T, with **C** denoting the stiffness tensor. Generally, this is a fairly small perturbation field required to enforce compatibility, i.e., ‖**C**^**−1**^**σ**‖ **≪** ‖**α**∆T‖. Consider, for example, a Taylor series expansion of the temperature field, i.e., *T* = *T*(**x = x**_**0**_) + (**x − x**_**0**_)*^T^***∇***T*∣_**x=x**_**0**__ + H. O. T., where H.O.T. denotes second-order and higher terms. The zeroth- and first-order terms trivially satisfy the strain compatibility equation for a homogeneous body, i.e., **∇ × ∇ ×** (*T*(**x=x**_**0**_) + (**x − x**_**0**_)*^T^***∇***T*∣_**x=x**_**0**__) = **0**, and, thus, only the higher-order terms generate stresses in an unconfined body. On the other hand, in the most extreme dynamic (instantaneous) thermal loading, i.e., *t* = 0^+^and τ_d_ = 0, the total strain is zero because of inertial confinement, i.e., **σ = −Cα**∆*T*. Under inertial confinement, stresses are affected by all the terms in the above Taylor series expansion, including (and especially) the zeroth- and first-order terms. As τ_d_ is increased, this inertial confinement is relaxed, with it vanishing entirely for τ_d_ ≫ τ_c_ at which point the stress field is determined entirely by the second-order and higher-order terms of **α**∆*T* that demand nonzero stress fields to satisfy strain compatibility, i.e., **∇ × ∇ ×** (**C**^**−1**^**σ + α∙** H. O. T. ) **= 0.**

### Directional effects

**Fig. 4.**
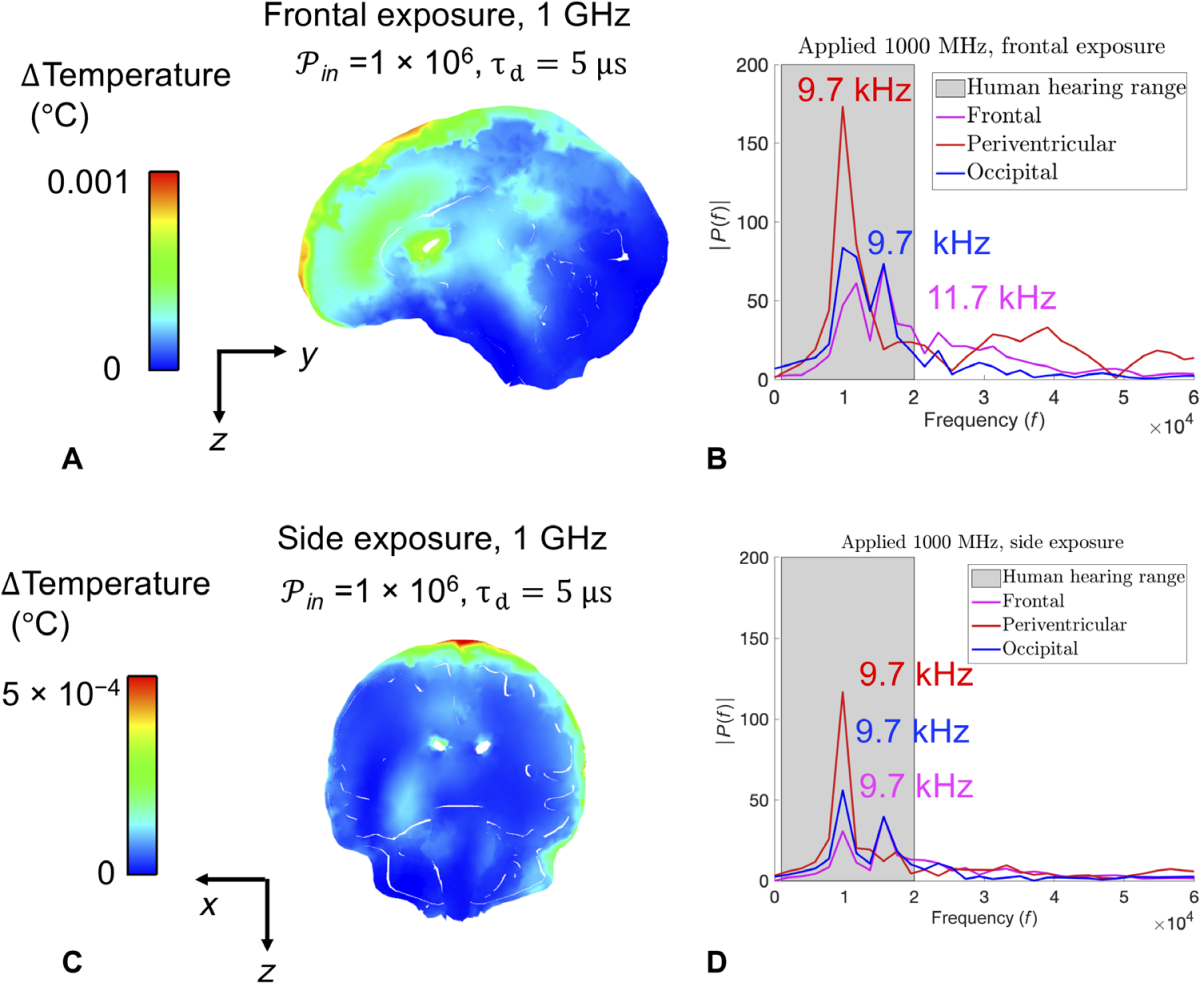
Comparison of directionality and its effect on frequency and amplitude. (**A**) Plot of final temperature distribution for τ_d_ = 5 μs, with 1 GHz of frequency frontal exposure (propagation toward the face of the individual, $P_{IN}$ = 1 × 10^6^ mW/cm^2^), and (**B**) Fourier transforms of pressure histories for frontal exposure scenario. (**C**) Plot of final temperature distribution for τ_d_ = 5 μs, with 1-GHz frequency side exposure (propagation toward the left side of the individual), and (**D**) Fourier transforms of pressure histories for side exposure scenario.

Two simulations were used to compare the possible changes in frequencies and amplitude when the body is positioned at different orientations relative to the source of EM radiation [e.g., frontal exposure versus side exposure, as shown in Fig. 4 (A and C)]. Both exposure scenarios use a 1-GHz exposure with the same power density and pulse duration ($P_{IN}$ = 1 × 10^6^ mW/cm^2^, τ_d_ = 5 μs). Consequently, the maximum temperatures of the brain and skin are slightly different in each case. This underscores the fact that different body orientations relative to EM field sources could create different hotspots, adding to the complexity in determining maximum brain temperatures.

A fast Fourier transform was used to compute the discrete Fourier transform of the pressure histories at the frontal, periventricular, and occipital locations in the brain. The resulting amplitude spectrum [*P*(*f*)] is shown in Fig. 4 (B and D). Although the dominant frequencies are almost identical, there is a change in amplitude. The peaks at 9.7 kHz are in agreement with previous research that microwave-induced cochlear microphonics in humans should be between 7 and 10 kHz and should not be influenced by the orientation of the body axis to the electrical field (*21*). In other words, the perceived pitch of a single pulse is determined primarily from the wave speed of tissues and the overall head dimensions. The loudness is correlated to the energy delivered to the brain tissue, which can change slightly based on the body’s orientation.

### Injury mechanisms

**Fig. 5.**
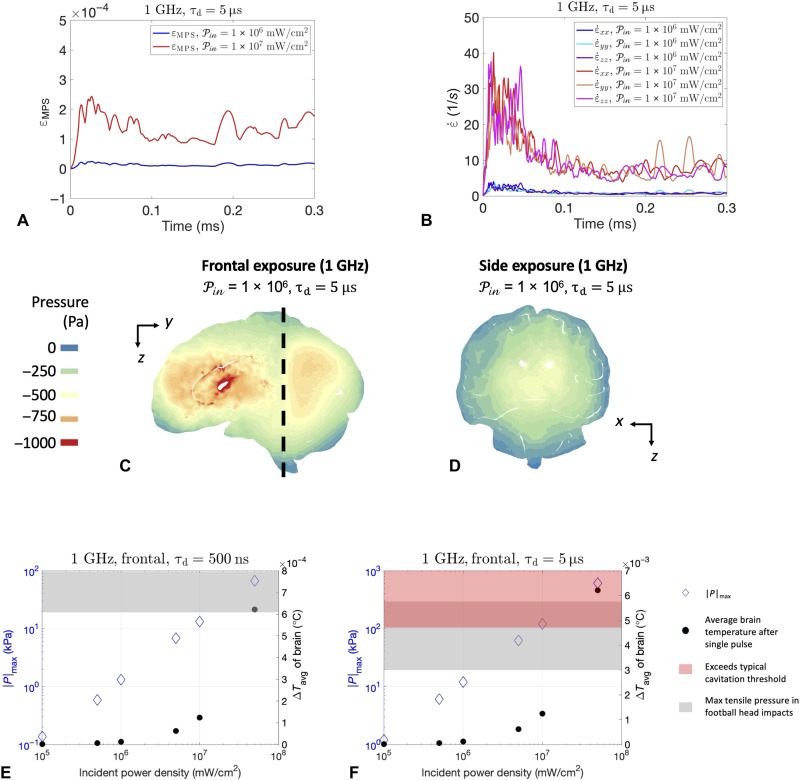
Summary of the mechanical response and comparison to TBI thresholds. (**A**) Global maximum of the maximum principal logarithmic strain (ε_MPS_). (**B**) Global maximum of strain rate over time ($\dot{\varepsilon }$) from exposure to a 1-GHz carrier wave (τ_d_ = 5 μs). Minimum pressure over time for frontal (**C**) and side exposure (**D**). (**E**) Plot of maximum tensile pressure and average brain temperature for various power densities for τ_d_ = 500 ns at 1 GHz. The average brain tissue shown includes the CSF. (**F**) Plot of maximum tensile pressure and average brain temperature for various power densities for τ_d_ = 5 μs at 1 GHz.

The most commonly used mechanics-based brain injury thresholds are typically strain based, because strain has experimentally been shown to cause mechanoporation and neuronal dysfunction (*22*, *23*). Although previous literature commonly cites an axonal strain injury threshold of 18% (*23*), more recent studies have shown that this injury threshold may be lower (e.g., 3 to 6%) for higher strain rates (*24*). Figure 5 (A and B) demonstrates that the maximum principal strains resulting from these exposure conditions are at least an order of magnitude smaller than injury thresholds; extreme power densities would be required to produce strain rates observed in typical impact or ballistic TBI events.

Although the simulations demonstrated small strains, it is evident that small (but rapidly applied) temperature increases from intense pulsed high peak power microwave exposures can induce high tensile stresses in the brain. A contour plot showing the minimum pressure (maximum tensile pressure) over the entire time history for frontal and side exposure of 1 GHz is shown in Fig. 5 (C and D, respectively). The results of Fig. 5 use $P_{IN}$ = 1 × 10^6^ mW/cm^2^, which is a reasonable upper limit given the publicly available literature on HPM technology (see Materials and Methods for more details). For both incident angles (frontal and side), large volumes of brain tissue are subjected to negative pressures. The regions with the largest tensile stresses depend on the direction of the applied EM exposure.

Figure 5 (E and F) shows the maximum tensile pressures for various power densities with a pulse duration of 500 ns and 5 μs, respectively. The intracranial temperatures attained after a single pulse are also shown. The simulations predict that exposures on the order of 10^6^ mW/cm^2^ would not result in tensile pressures that are likely to induce cavitation; however, the pressures are non-negligible (tens of kilopascals). Figure 5E (τ_d_ = 500 ns) shows that, for power densities $P_{IN}=$ 1 × 10^7^ mW/cm^2^, the maximum tensile pressures are comparable to the tensile pressures seen in typical head impacts in National Football League (NFL) football players (20 to 120 kPa) (*25*). For the longer pulse duration shown in Fig. 5F (τ_d_ = 5 μs), the maximum tensile pressures for $P_{IN}=$ 1 × 10^7^ mW/cm^2^ exceed cavitation. For power densities above 1.5 × 10^6^ mW/cm^2^, it is evident that tensile pressures are comparable to football head impacts (*25*). It is possible that slightly higher tensile pressures can be achieved with longer pulse durations (e.g., τ_d_ = 50 μs), but, as previously mentioned, increasing the pulse duration by too much (greater than the intrinsic time scales of the system) can diminish the formation of stress waves.

## DISCUSSION

Another possible injury mechanism is cavitation, which refers to the unstable expansion of bubbles from a liquid following a rapid change in pressure. Although cavitation is believed to be a possible injury mechanism in TBI, its behavior in brain tissue is far from fully characterized (*26*). The rapid expansion and collapse of cavitation bubbles may result in large deformation of the surrounding brain tissue. Commonly cited thresholds for brain tissue cavitation are negative pressures of roughly −100 to −150 kPa (*26*–*28*). The uncertainty in the brain tissue cavitation threshold is due to the fact that the critical pressure is a function of possible nucleation bubble sizes (see details in Materials and Methods), as well as the fact that critical pressure and nucleation in dynamic systems are still active areas of research. Although the formation mechanism and sites for cavitation “seed” bubbles/nuclei in brain tissue remain unknown (*29*), it has been suggested that nanobubbles containing dissolved gas such as oxygen within the fluid spaces of the brain could act as sites of nucleation (*30*).

In our simulations of both loading directions, we see the stress-focusing effect causing large negative pressures in the periventricular, diencephalon, and midbrain regions (encompassing the inferior colliculus and other areas important to eye movement and hearing response). Although the brain is believed to naturally vary in temperature between 1° and 3°C (*31*), these changes occur relatively slowly through changes in cerebral blood flow. This study has shown that, by applying a small temperature increase (<0.0005°C) in a very short amount of time (less than several microseconds), potentially injurious stress waves are created. The temperatures presented in this study are well below the threshold (8°C) known to cause unconsciousness in rodents following a single HPM pulse (*32*).

This study has specifically discussed the transient thermomechanical response to a single high peak power pulse at extremely short time scales. It could be possible to generate an injurious response with lower power densities at an extremely high repetition rate, resulting in the same temperature load (~0.0005°C in <5 μs). The behavior of cyclic loading and rapid fluctuations in pressure at longer time scales could potentially induce different injury thresholds that are unknown at this time. High peak power RF pulses result in a loading regime that is different from conventional blunt impact forces associated with TBI. The mechanical and biological response of brain tissue at this extremely high-rate regime requires further investigation.

We did not thoroughly delve into the details of multiple pulses or various repetition rates at longer time scales. At longer time scales (i.e., seconds and minutes), there are also effects of thermoregulation because the body would attempt to lower the temperature in the brain through changes in blood flow and CSF circulation (*33*, *34*). The computational model does not include vasculature, which could alter the initial temperature distribution and potentially produce stress concentrations similar to what is seen in the brain tissue surrounding the ventricles. Elucidation of the mechanical thresholds for mild TBI is still an active area of research, and the findings of this study should not be considered as clinically conclusive evidence.

Nevertheless, the simulations here have shown that exceptionally intense HPM exposures with incident power densities greater than 1.5 × 10^6^ mW/cm^2^ (at short pulse durations) may generate intracranial stresses that are similar (±∼20 to 200 kPa) in comparison to typical TBI events (sports, vehicle accidents, ballistic impact, etc.). For sufficiently short microwave pulse durations (<τ_c_), large tensile stresses are created in the deep regions of the brain. These high tensile stresses might possibly result in cavitation (in addition to the previously well-known auditory effect) for extreme exposures (incident power densities of 10^7^ mW/cm^2^). The power densities required to cause cavitation are physically possible (below the theoretical upper limit of dielectric breakdown of microwaves in air) but large enough to cause disturbances (possibly damage) to electronic devices subjected to the same power densities (*35*). The stresses resulting from power densities of 10^6^ mW/cm^2^ are on the order of tens of kilopascals (for comparison, at the lower end of football impacts). A repeated pulsed exposure to any of these injurious power densities for long periods of time (e.g., several minutes) would likely induce noticeable thermal sensations of the skin, even at very low pulse repetition frequencies. However, a single intense pulse could be injurious.

For frequencies between 400 MHz to 2 GHz, the IEEE C95.1 RF exposure guidelines limit the exposure reference limit (ERL) to *f*_mhz_/200 (W/m^2^) over an averaging time of 30 min. For 1-GHz exposures, the IEEE C95.1 ERL of 5 W/m^2^ over 30 min would equate to an average energy density of 9000 J/m^2^. Our computational model shows that, for sufficiently high incident power densities, a single pulse could potentially result in biologically meaningful pressures. For example, large pressures may occur following 1-GHz frequency, a pulse duration of 5 μs, and incident power densities of at least 1.5 × 10^7^ W/m^2^. The energy density associated with a such a pulse would be equal to $P_{IN}\times \tau_{d}$ or 75 J/m^2^ (significantly less than the ERL standard).

Note that the proposed HPM power densities in this study are extremely large and several orders of magnitude larger than power densities typically experienced by the public. As an illustrative example, at around 200 feet from a cell phone base station, a person will be exposed to a power density of only 0.001 mW/cm^2^ or less (*36*). This study establishes a testable hypothesis between potential neurocognitive effects and the thermoelastic mechanism from HPM systems. To date, however, adverse effects from HPM systems have not been established in the scientific literature.

## MATERIALS AND METHODS

### EM plane wave simulations

Multiphysics computational approaches are often used in combination with realistic digital anatomical body models to simulate EM dosimetry and the resulting changes in tissue temperature. Here, this approach is used to (i) simulate the absorption of microwave energy at a range of frequencies, (ii) determine optimal RFs for mechanical simulations based upon volume-averaged changes in brain temperature, and (iii) capture absorption and heating patterns at these conditions to serve as inputs into mechanical simulations. These mechanical simulations will be detailed in the “Thermoelastic expansion simulations” section.

To create the head model used in these simulation activities, a T1-weighted sagittal sequence image of the whole brain (male subject) was obtained by a magnetization-prepared rapid acquisition gradient echo (MPRAGE) at 3 T with a Siemens Tim Trio magnetic resonance imaging (MRI) scanner [see (*37*) for scanning parameters]. MRI data were segmented and converted into a 3D surface geometry with Amira software (Thermo Fisher Scientific) (*38*). The skin of the human head model was then attached to the skin geometry of a digital male body (Zygote Media Group). The head model contained detailed 3D geometries, while the remaining volume of the body was assumed to be a homogeneous muscle mass. Although the MRI data were obtained at a resolution of 1 mm, the 3D geometry was voxelized at a resolution of 2 mm for the FDTD calculations and assigned the dielectric material properties shown in table S1.

The developed head and body model were then used within the custom FDTD software to calculate SAR values. The FDTD method is a commonly used numerical technique that provides an approximate solution to Maxwell’s equations (*39*) and staggers the electric (**E**) and magnetic (**H**) fields about box-shaped cells so that the spatial derivatives of the EM field can be accurately solved with a centered difference scheme. As a rule of thumb, there should be at least 10 cells per wavelength (higher frequency simulations require higher mesh resolution).

Time is quantized into steps where each time step represents the time required for the field to travel from one cell to the next. Similarly, the time derivatives of **E** and **H** are evaluated at alternate half-time steps, until a state of convergence has been reached. One disadvantage of the FDTD method is that the cells surrounding the body of interest must also be modeled and that the fields on the outer boundary of the grid could be updated inaccurately. To address this issue, outer boundaries are treated with a perfectly matched layer condition that mimics an absorbing material. A more detailed explanation of the FDTD algorithm, as well as validation against analytical solutions derived from Mie theory, is provided by Adams *et al*. (*15*). SAR is defined as an incremental power *dP* absorbed by an incremental mass of tissue *dm* (SAR = *dP*/*dm*). Following simple mathematical operations and substitution of terms with Ohm’s law, one can obtain the following expression for SAR at each voxel in the frequency domain (*16*)

$$\text{SAR}=\frac{\sigma {|E|}^{2}}{\rho }$$

Where *E* is the root mean square of the electric field and ρ is the tissue density. For sufficiently short RF pulses, thermal diffusivity can be negligible [assuming a brain tissue thermal diffusivity of 2 × 10^−7^ m^2^/s (*40*)], and the change in tissue temperature at any position is linearly related to the SAR at that position through Eq. 1.

The SAR analysis was limited to the head and neck, because this region of the model included the most anatomical detail. It is possible that additional “hotspots” can be created in other regions of the body [e.g., near the wrists or ankles (*41*)] due to the exposure conditions, geometry, and heat dissipation mechanisms. However, our thermal analysis suggests that any such hotspots would remain well below thermal sensation levels for worst-case exposures to even an exceptionally intense single-pulse exposure condition. Because single-voxel SAR data are prone to numerical artifact due to the staircasing approximation of curved surfaces, we use the peak 10-g averaged SAR of the skin and intracranial regions (as shown in Fig. 2B).

The changes in tissue temperature calculated by Eq. 1 are used as inputs into the thermoelastically driven mechanics simulations. The temperature values calculated for each voxel in the RF/microwave simulations provide the final temperature at the end of the applied pulse duration (denoted here as τ_d_). The temperature is approximated as a linear ramp to the final temperature at time *t* = τ_d_ as shown in Fig. 1C. For the case of frontal exposure to a vertically polarized plane wave (with frequencies of 800 MHz to 2 GHz), the highest intracranial temperatures are shown near the frontal lobe and ventricles.

### Thermoelastic expansion simulations

The previously described segmented surface geometries of the skull (cranial vault), CSF, white matter, gray matter, ventricles, and brainstem were imported into the meshing software Ansys ICEM CFD (Ansys, Canonsburg, PA). The FEM only encompasses the skull bone and intracranial contents. It is assumed that the facial bone, skin, and sinuses outside of the skull do not influence the mechanical response of the brain tissue. Although the skin, facial bones, and sinuses are not modeled in the FEM, they are previously incorporated in the FDTD simulations to accurately capture the RF pulse–induced heating of the brain, CSF, and skull.

Because of the elongated structure of the brainstem and its proximity to noise-generating sources such as the heart and lungs, it is difficult to segment the gray matter nuclei [which have an average cross-sectional diameter of a few millimeters (*42*)]. For these reasons, the white and gray matter regions of the brainstem were combined and assigned uniform (white matter) material properties.

Finite element simulations were performed with a Lagrangian, 3D explicit dynamics code (Sierra, Sandia National Laboratories). The shared nodes at the material interfaces were prescribed a “tied contact” formulation. The skull, CSF, white matter, gray matter, and ventricles were assigned the material properties provided in table S2. To ensure that adequate material properties and contact definitions were prescribed, a validation study of the mechanical response was previously performed (*18*) by comparing simulations of the model to experimental data from cadaveric head impacts found in the literature (*43*).

The spatial resolution of the FDTD model was 2 mm, whereas the FEM has various element sizes (with an average size closer to 1 mm in the brain tissue). Each node of the FEM was assigned an increase in temperature based on the SAR value of the voxel in closest proximity. A temperature history (with length of time corresponding to the desired pulse duration) was applied to all voxels comprising the white matter, gray matter, and CSF. Because bone has a significantly smaller coefficient of thermal expansion (α ∼ 0.27 × 10^−4^/°C) (*44*), it was assumed that the expansion of the bone, and therefore the skin/scalp, did not affect the thermoelastic expansion of brain tissue and CSF. This assumption is further justified by the smaller electrical conductivity and SAR values in bone relative to soft tissues. A summary of finite element results from this study and A.M.D. and J.W.W.’s study (*18*) is shown in table S3.

### Assumed power densities

Although we are interested in the pressure fields produced by large peak power density exposures, reasonable constraints should be placed upon the power densities considered. These constraints are based on engineering estimates of microwave hardware capabilities. Therefore, we limit our analysis to (i) power densities below dielectric breakdown in air and (ii) power densities that are achievable at range with known existing technologies.

Previous studies have suggested that the MAE is typically achieved with pulse durations ranging anywhere from 0.5 to 500 μs and pulse repetition rates typically ranging from 1 to 1000 Hz (*45*). These studies reveal that the MAE threshold depends on the energy in a single pulse (not the average power density) for sufficiently short pulses [e.g., 32 μs in (*46*)], and peak power densities of 10^2^ to 10^5^ mW/cm^2^ have been known to cause auditory effects in human participants (*45*).

While the peak power densities used within this simulation study are large, they are achievable with known microwave hardware. For example, to produce a power density of 1 × 10^6^ mW/cm^2^ at 25 m away from a 40-dBi antenna, a microwave source would require approximately 8 MW of power per pulse. This is within the capabilities of some commercial and military systems, and we therefore consider this as a relevant approximation for the simulations here. However, we also consider some more extreme conditions in the final analysis summary for scaling purposes against known mechanical TBI thresholds.

## Supplementary Materials

### This PDF file includes:

Tables S1 to S3 References
