# Supplementary material for: Computational modeling investigation of pulsed high peak power microwaves and the potential for traumatic brain injury
Source: Sci Adv. 2021 Oct 29;7(44):eabd8405. doi: 10.1126/sciadv.abd8405 (PMC8555891; doi:10.1126/sciadv.abd8405)
Supplement: Supplementary file 1 — Tables S1 to S3 References [file sciadv.abd8405_sm.pdf]

Science Advances
AAAS

Supplementary Materials for

Computational modeling investigation of pulsed high peak power microwaves and the potential for traumatic brain injury

Amy M. Dagro*, Justin W. Wilkerson, Thaddeus P. Thomas, Benjamin T. Kalinosky, Jason A. Payne

*Corresponding author. Email: amy.m.dagro.civ@mail.mil

Published 29 October 2021, *Sci. Adv.* **7**, eabd8405 (2021)
DOI: 10.1126/sciadv.abd8405

This PDF file includes:

Tables S1 to S3
References

Part	ρ (kg/m^3)	ϵ_R	σ_c (S/m)	Ref.
Air	1.16 x 10^1	1.0	0.0	[47]
Corpus callosum	1.04 x10^3	3.9 x 10^1	5.6 x 10^{-1}	[47]
Cerebellum gray matter	1.04 x 10^3	5.3 x 10^1	9.0 x 10^{-1}	[47]
Cerebellum white matter	1.04 x 10^3	3.9 x 10^1	5.6 x 10^{-1}	[47]
Ventricles	1.00 x 10^3	6.8 x 10^1	2.4	[47]
Cerebrospinal fluid	1.00 x 10^3	6.8 x 10^1	2.4	[47]
Gray matter	1.04 x 10^3	5.3 x 10^1	9.0 x 10^{-1}	[47]
Sinus	1.16 x 10^1	1.0	0.0	[47]
Skull (cranial bone, facial bone, jaw)	1.91 x 10^3	1.3 x 10^1	1.3 x 10^{-1}	[47]
Brain stem	1.04 x 10^3	5.3 x 10^1	9.0 x 10^{-1}	[47]
White matter	1.04 x 10^3	3.9 x 10^1	5.6 x 10^{-1}	[47]
Skin	1.11 x 10^3	4.2 x 10^1	8.3 x 10^{-1}	[47]
Homogeneous body tissue (muscle)	1.10 x 10^3	5.5 x 10^1	9.1 x 10^{-1}	[47]

Table S1. Material properties used in the FDTD simulations. ε_R is the relative permittivity and σ_c is the conductivity. All material properties taken from [47].

Part	Material model	Properties	Ref.
Brain white matter (WM)	Mooney Rivlin w/ Prony series	g_1=0.316, g_2=0.428, $\tau_1 = 3\ s$, $\tau_2 = 0.19\ s$, C_{10}=130 Pa, C_{01}=135 Pa,<br>$\alpha = 6.7$ x 10^{-4} / °C<br>K=2 GPa<br>$\rho = 1040$ kg/m^3<br>c_p =3500 J/kg/ °C	[48]<br>[49]<br>[50]<br>[51]<br>[52]<br>[47]
Brain gray matter (GM)	Mooney Rivlin w/ Prony series	g_1=0.335, g_2=0.461,$\tau_1 = 2.4\ s$, $\tau_2 = 0.15\ s$, C10=130 Pa, C01=135 Pa,<br>$\alpha = 2$ x 10^{-4} / °C<br>K=2 GPa<br>$\rho = 1040$ kg/m^3<br>c_p =3700 J/kg/ °C	[48]<br>[49]<br>[50]<br>[51]<br>[52]<br>[47]
Skull	Linear elastic	E=1 GPa<br>$\nu = 0.19$<br>$\rho = 1134$ kg/m^3<br>c_p =1650 J/kg/ °C<br>α =2 x10^{-5} / °C	[53]<br>[54]<br>[55]<br>[47]<br>[56]
CSF and ventricles	Linear elastic	K=2 GPa<br>G=50 Pa<br>$\alpha = 0.6$ x 10^{-4} / °C<br>$\rho = 1020$ kg/m^3<br>c_p =4000 J/kg/ °C	[57]<br>[58]<br>[44]<br>[52]<br>[47]

Table S2. Thermomechanical material properties used in FEM simulations.

Geometry	Loading	τ_d (μs)	ΔT_{avg} (°C)	ΔT_{max} (°C)	$\lvert P \rvert_{max}$ (MPa)	Efficiency factor	Ref.
Human	800 MHz	5	0.071	1.07	10.8	6340%	This work

	frontal exposure						
Human	800 MHz frontal exposure	500	0.071	1.07	0.125	73.9%	This work
Human	1 GHz frontal exposure	5	0.044	0.668	6.82	6440%	This work
Human	1 GHz side exposure	5	0.038	0.317	3.85	4230%	This work
Human	1 GHz frontal exposure	1	0.002	0.073	0.31	7180%	This work
Sphere	Uniform heating $1\,°C$	5	1.00	1.00	18.7	472%	[18]
Sphere	Uniform heating $8\,°C$	0.5	8.00	8.00	149	469%	[18]
Sphere	Surface heating $8\,°C$	0.5	2.83	8.00	443	3590%	[18]
Sphere	Core heating $8\,°C$	0.5	0.103	8.00	7.34	1790%	[18]

Table S3. Summary of results from simulations presented in this work and Ref. (14). ΔT_{avg} and ΔT_{max} are the average and maximum temperature rises of the brain (white and gray matter). $|P|_{max}$ is the maximum local tensile pressure experienced during the simulation. The efficiency factor is given as $|P|_{max}/3\alpha K\Delta T_{avg}$.

REFERENCES AND NOTES

1. SCC39, IEEE Standard for Safety Levels with Respect to Human Exposure to Electric, Magnetic, and Electromagnetic Fields, 0 Hz to 300 GHz (IEEE, 2019).

2. K. R. Foster, R. Glaser, Thermal mechanisms of interaction of radiofrequency energy with biological systems with relevance to exposure guidelines. *Health Phys.* **92**, 609–620 (2007).

3. J. Vila, Intermediate and radiofrequency sources and exposures in everyday environments, in *Bioengineering and Biophysical Aspects of Electromagnetic Fields* (CRC Press, 2018), p. 55.

4. K. R. Foster, Thermal and nonthermal mechanisms of interaction of radio-frequency energy with biological systems. *IEEE Trans. Plasma Sci.* **28**, 15–23 (2000).

5. K. H. Schoenbach, C. C. Tseng, Subcellular responses to narrowband and wideband radiofrequency radiation (Old Dominion University Research Foundation, 2008).

6. T. Kotnik, W. Frey, M. Sack, S. H. Meglič, M. Peterka, D. Miklavčič, Electroporation-based applications in biotechnology. *Trends Biotechnol.* **33**, 480–488 (2015).

7. A. Frey, Auditory system response to radio frequency energy. Technical note. *Aerosp. Med.* **32**, 1140–1142 (1961).

8. A. Frey, Human auditory system response to modulated electromagnetic energy. *J. Appl. Physiol.* **17**, 689–92 (1962).

9. E. M. Taylor, B. T. Ashleman, Analysis of central nervous system involvement in the microwave auditory effect. *Brain Res.* **74**, 201–208 (1974).

10. K. R. Foster, E. D. Finch, Microwave hearing: Evidence for thermoacoustic auditory stimulation by pulsed microwaves. *Science* **185**, 256–258 (1974).

11. A. Frey, Electromagnetic field interactions with biological systems. *FASEB J.* **7**, 272–281 (1993).

12. J. Lin, *Microwave Auditory Effects and Applications* (Springfield, IL: Thomas, 1978).

13. T. G. Raslear, Y. Akyel, F. Bates, M. Belt, S. T. Lu, Temporal bisection in rats: The effects of high-peak-power pulsed microwave irradiation. *Bioelectromagnetics* **14**, 459–478 (1993).

14. Y. Watanabe, T. Tanaka, M. Taki, S. Watanabe, FDTD analysis of microwave hearing effect. *IEEE Trans. Microw. Theory Tech.* **48**, 2126–2132 (2000).

15. S. Adams, J. Payne, L. Harris, J. Ziriax, "Modeling Exposure to Electromagnetic Fields with Realistic Anatomical Models: The Brooks Finite Difference Time Domain (FDTD)," (Naval Health Research Center, San Diego, 2008).

16. J. C. Lin, *Electromagnetic Fields in Biological Systems* (CRC press, 2011).

17. R. B. Hetnarski, M. Reza Eslami, *Thermal Stresses—Advanced Theory and Applications* (Springer, 2009).

18. A. Dagro, J. Wilkerson, A computational investigation of strain concentration in the brain in response to a rapid temperature rise. *J. Mech. Behav. Biomed. Mater.* **115**, 104228 (2021).

19. Y. Chen, M. Ostoja-Starzewski, MRI-based finite element modeling of head trauma: Spherically focusing shear waves. *Acta Mechanica* **213**, 155–167 (2010).

20. B. Ravaji, V. Alí-Lagoa, M. Delbo, J. W. Wilkerson, Unraveling the mechanics of thermal stress weathering: Rate-effects, size-effects, and scaling laws. *J. Geophys. Res.* **124**, 3304–3328 (2019).

21. C.-K. Chou, A. W. Guy, R. Galambos, Characteristics of microwave-induced cochlear microphonics. *Radio Sci.* **12**, 221–227 (1977).

22. M. C. LaPlaca, M. C. Lessing, Assessment of membrane permeability after traumatic brain injury, in *Animal Models of Acute Neurological Injuries II* (Humana Press, 2012), pp. 275–298.

23. A. C. Bain, D. F. Meaney, Tissue-level thresholds for axonal damage in an experimental model of central nervous system white matter injury. *J. Biomech. Eng.* **122**, 615–622 (2000).

24. E. W. Vogel III, M. B. Panzer, F. N. Morales, N. Varghese, C. R. Bass, D. F. Meaney, B. Morrison III, Direct observation of low strain, high rate deformation of cultured brain tissue during primary blast. *Ann. Biomed. Eng.* **48**, 1196–1206 (2020).

25. L. Zhang, K. H. Yang, A. I. King, A proposed injury threshold for mild traumatic brain injury. *J. Biomech. Eng.* **126**, 226–236 (2004).

26. C. Franck, Microcavitation: The key to modeling blast traumatic brain injury? *Concussion* **2**, CNC47 (2017).

27. J. Goeller, A. Wardlaw, D. Treichler, J. O'Bruba, G. Weiss, Investigation of cavitation as a possible damage mechanism in blast-induced traumatic brain injury. *J. Neurotrauma* **29**, 1970–1981 (2012).

28. R. S. Salzar, D. Treichler, A. Wardlaw, G. Weiss, J. Goeller, Experimental investigation of cavitation as a possible damage mechanism in blast-induced traumatic brain injury in post-mortem human subject heads. *J. Neurotrauma* **34**, 1589–1602 (2017).

29. S. Canchi, K. Kelly, Y. Hong, M. A. King, G. Subhash, M. Sarntinoranont, Controlled single bubble cavitation collapse results in jet-induced injury in brain tissue. *J. Mech. Behav. Biomed. Mater.* **74**, 261–273 2017.

30. U. Adhikari, A. Goliaei, M. L. Berkowitz, Nanobubbles, cavitation, shock waves and traumatic brain injury. *Phys. Chem. Chem. Phys.* **18**, 32638–32652 (2016).

31. H. Wang, B. Wang, K. P. Normoyle, K. Jackson, K. Spitler, M. F. Sharrock, C. M. Miller, C. Best, D. Llano, R. Du, Brain temperature and its fundamental properties: A review for clinical neuroscientists. *Front. Neurosci.* **8**, 307 (2014).

32. A. W. Guy, C.-K. Chou, Effects of high-intensity microwave pulse exposure of rat brain. *Radio Sci.* **17**, 169S–178S (1982).

33. H. Wang, M. Kim, K. P. Normoyle, D. Llano, Thermal regulation of the brain—An anatomical and physiological review for clinical neuroscientists. *Front. Neurosci.* **9**, 528 (2016).

34. S. Kodera, J. Gomez-Tames, A. Hirata, Temperature elevation in the human brain and skin with thermoregulation during exposure to RF energy. *Biomed. EngineeringOnline* **17**, 1 (2018).

35. J. Benford, J. A. Swegle, E. Schamiloglu, *High Power Microwaves* (CRC Press, 2015).

36. B. B. Levitt, H. Lai, Biological effects from exposure to electromagnetic radiation emitted by cell tower base stations and other antenna arrays. *Environ. Rev.* **18**, 369–395 (2010).

37. D. S. Bassett, J. A. Brown, V. Deshpande, J. M. Carlson, S. T. Grafton, Conserved and variable architecture of human white matter connectivity. *Neuroimage* **54**, 1262–1279 (2011).

38. R. H. Kraft, P. J. Mckee, A. M. Dagro, S. T. Grafton, Combining the finite element method with structural connectome-based analysis for modeling neurotrauma: Connectome neurotrauma mechanics. *PLOS Comput. Biol.* **8**, e1002619 (2012).

39. K. Yee, Numerical solution of initial boundary value problems involving Maxwell's equations in isotropic media. *IEEE Trans. Antennas Propag.* **14**, 302–307 (1966).

40. K. G. Kastella, J. R. Fox, The dynamic response of brain temperature to localized heating. *Biophys. J.* **11**, 521–539 (1971).

41. P. R. Wainwright, The relationship of temperature rise to specific absorption rate and current in the human leg for exposure to electromagnetic radiation in the high frequency band. *Phys. Med. Biol.* **48**, 3143–3155 (2003).

42. R. Sclocco, F. Beissner, M. Bianciardi, J. R. Polimeni, V. Napadow, Challenges and opportunities for brainstem neuroimaging with ultrahigh field MRI. *Neuroimage* **168**, 412–426 (2018).

43. W. N. Hardy, M. J. Mason, C. D. Foster, C. S. Shah, J. M. Kopacz, K. H. Yang, A. I. King, A study of the response of the human cadaver head to impact. *Stapp Car Crash J.* **51**, 17–80 (2007).

44. F. A. Duck, *Physical Properties of Tissues: A Comprehensive Reference Book* (Academic Press, 2013).

45. J. A. Elder, C. K. Chou, Auditory response to pulsed radiofrequency energy. *Bioelectromagnetics* **24**, S162-S173 (2003).

46. A. W. Guy, C. K. Chou, J. C. Lin, D. Christensen, Microwave-induced acoustic effects in mammalian auditory systems and physical materials. *Ann. N. Y. Acad. Sci.* **247**, 194–218 (1975).

47. P. A. Hasgall, F. Di Gennaro, C. Baumgartner, E. Neufeld, B. Lloyd, M. C. Gosselin, D. Payne, A. Klingenböck, N. Kuster, "IT'IS Database for thermal and electromagnetic parameters of biological tissues," (2018); itis.swiss/database.

48. M. T. Prange, S. S. Margulies, Regional, directional, and age-dependent properties of the brain undergoing large deformation. *J. Biomech. Eng.* **124**, 244–252 (2002).

49. S. Budday, G. Sommer, C. Birkl, C. Langkammer, J. Haybaeck, J. Kohnert, M. Bauer, F. Paulsen, P. Steinmann, E. Kuhl, G. A. Holzapfel, Mechanical characterization of human brain tissue. *Acta Biomater.* **48**, 319–340 (2017).

50. J. Mendez, A. Keys, J. T. Anderson, F. Grande, Density of fat and bone mineral of the mammalian body. *Metabolism* **9**, 472–477 (1960).

51. J. H. McElhaney, J. W. Melvin, V. L. Roberts, H. D. Portnoy, Dynamic characteristics of the tissues of the head, in *Perspectives in Biomedical Engineering* (Palgrave Macmillan, 1973), pp. 215–222.

52. T. W. Barber, J. A. Brockway, L. S. Higgins, The density of tissues in and about the head. *Acta Neurol. Scand.* **46**, 85–92 (1970).

53. A. D. Brown, K. A. Rafaels, T. Weerasooriya, "Microstructural and rate-dependent shear response of human skull bones" (CCDC Army Research Laboratory, Aberdeen Proving Ground, mD, 2020).

54. J. H. McElhaney, J. L. Fogle, J. W. Melvin, R. R. Haynes, V. L. Roberts, N. M. Alem, Mechanical properties of cranial bone. *J. Biomech.* **3**, 495–511 (1970).

55. S. L. Alexander, K. Rafaels, C. A. Gunnarsson, T. Weerasooriya, Structural analysis of the frontal and parietal bones of the human skull. *J. Mech. Behav. Biomed. Mater.* **90**, 689–701 (2019).

56. S. Pal, S. Saha, Coefficient of thermal expansion of bone, in *Biomechanics* (John Wiley and Sons Inc., 1989), pp. 52–60.

57. S. Ganpule, N. P. Daphalapurkar, M. P. Cetingul, K. T. Ramesh, Effect of bulk modulus on deformation of the brain under rotational accelerations. *Shock Waves* **28**, 127–139 (2018).

58. A. I. King, *The Biomechanics of Impact Injury* (Chalm, Switzerland, Springer, 2018).
